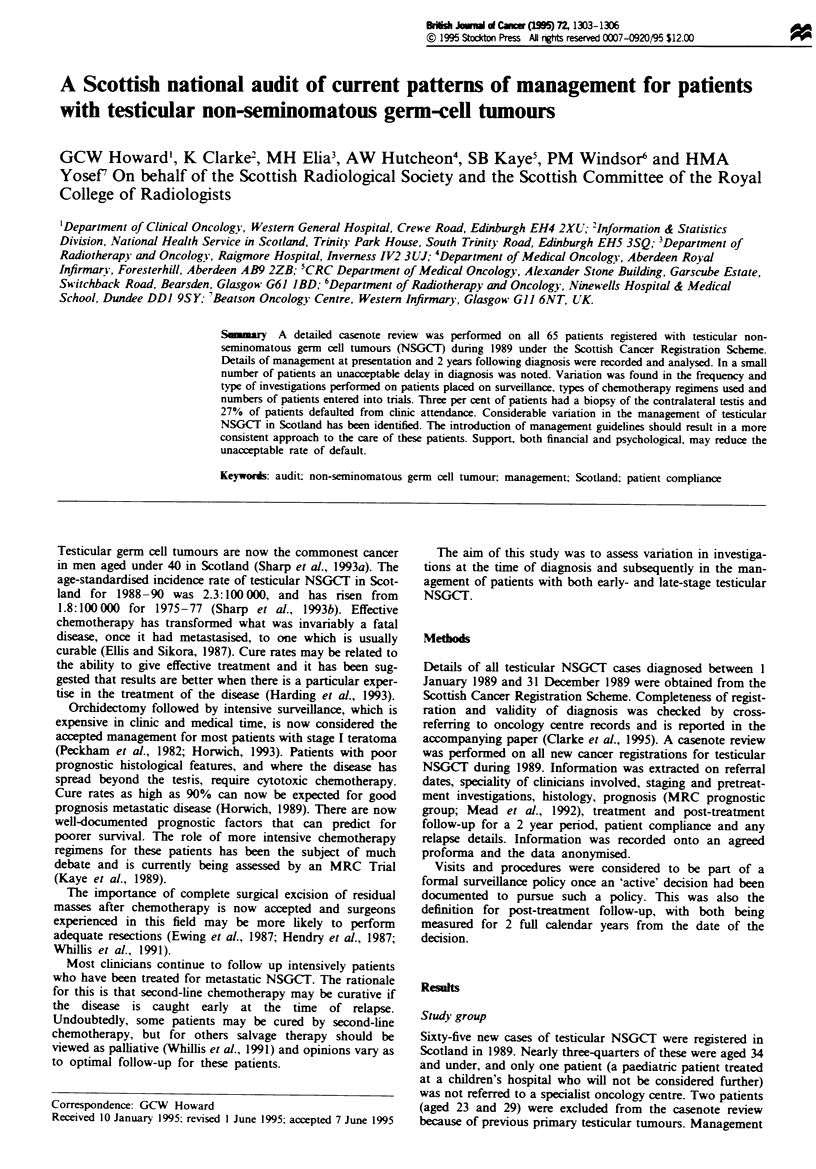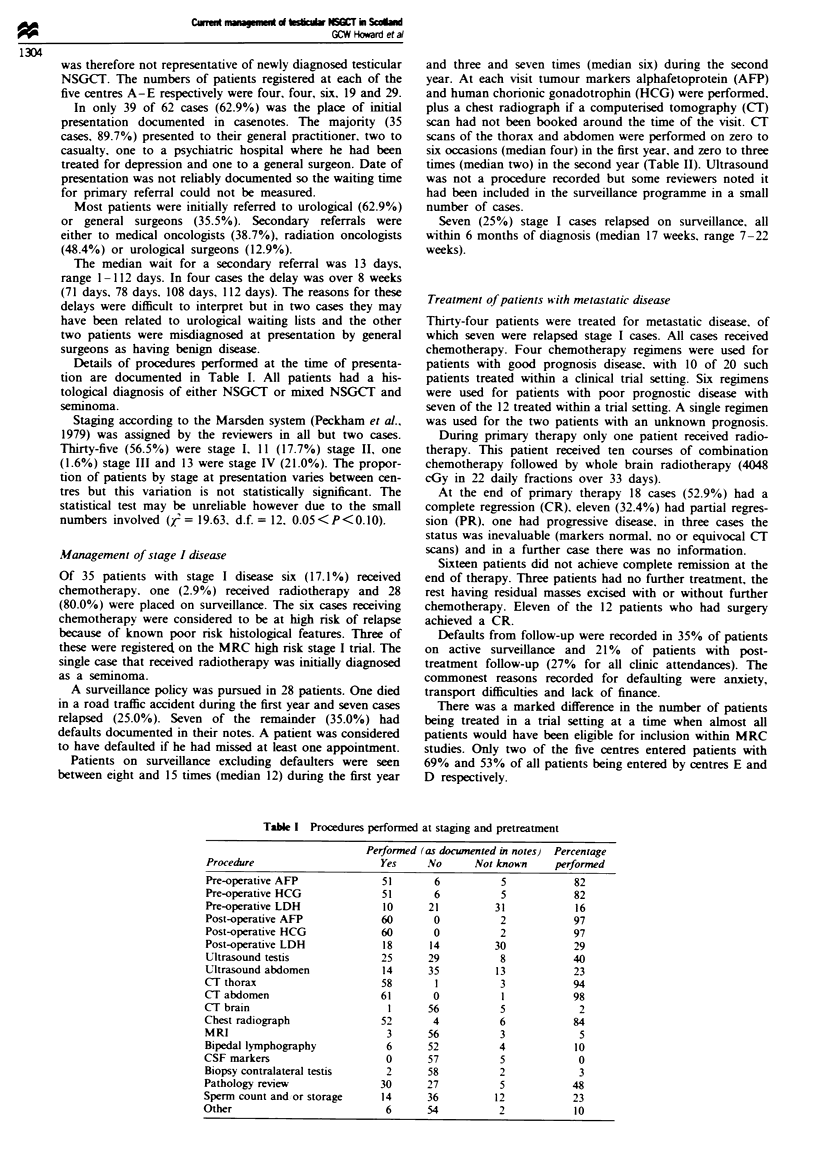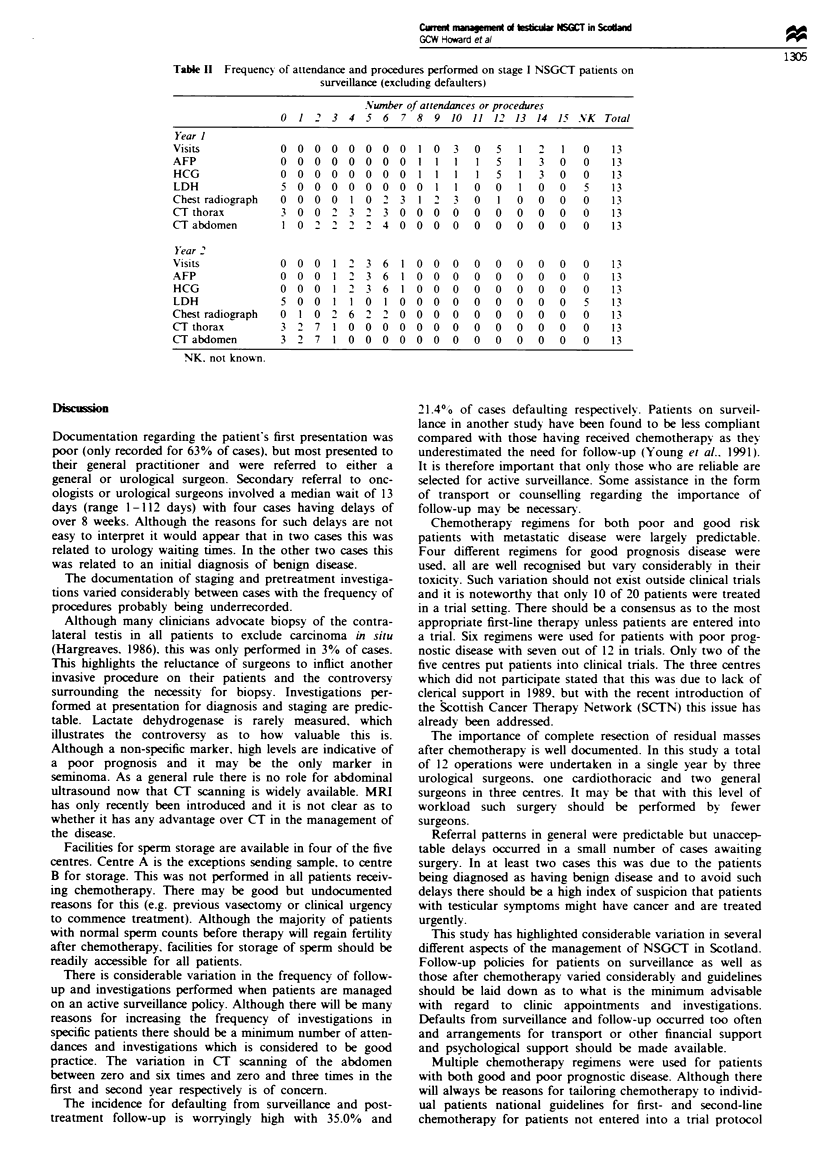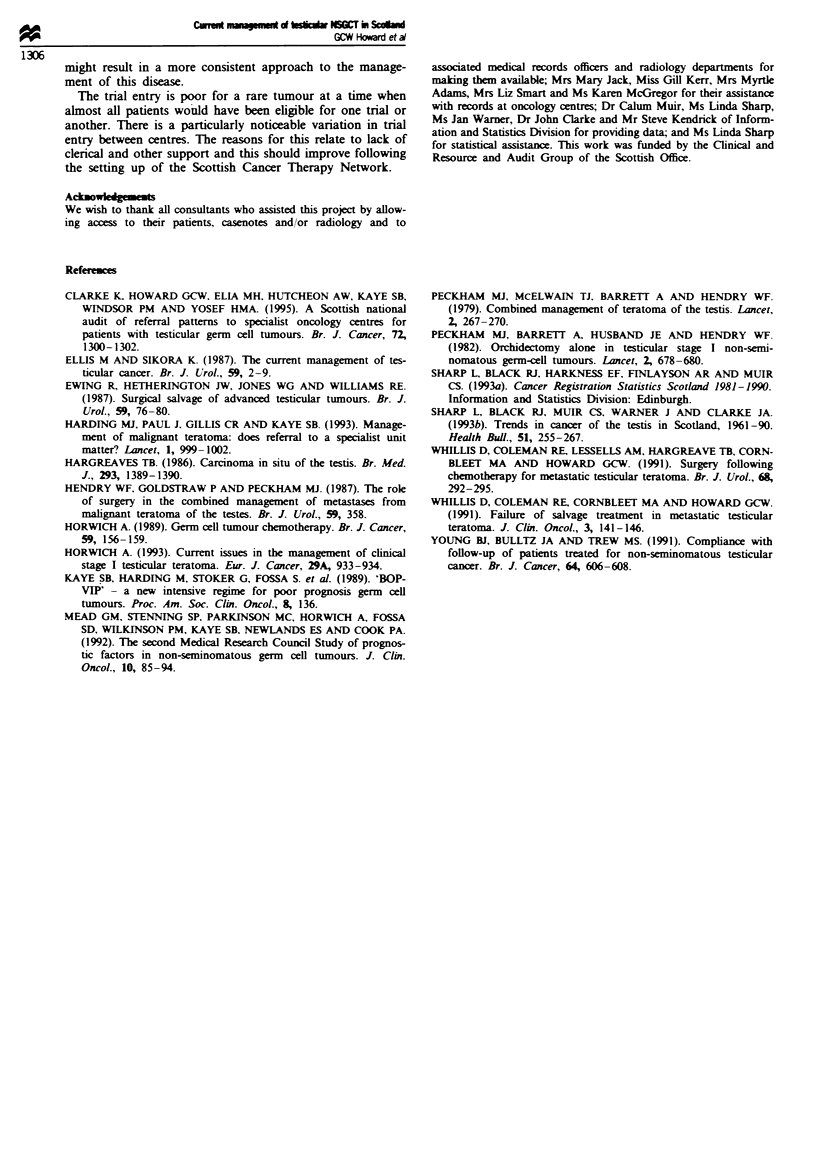# A Scottish national audit of current patterns of management for patients with testicular non-seminomatous germ-cell tumours. The Scottish Radiological Society and the Scottish Committee of the Royal College of Radiologists.

**DOI:** 10.1038/bjc.1995.505

**Published:** 1995-11

**Authors:** G. C. Howard, K. Clarke, M. H. Elia, A. W. Hutcheon, S. B. Kaye, P. M. Windsor

**Affiliations:** Department of Clinical Oncology, Western General Hospital, Edinburgh, UK.

## Abstract

A detailed casenote review was performed on all 65 patients registered with testicular non-seminomatous germ cell tumours (NSGCT) during 1989 under the Scottish Cancer Registration Scheme. Details of management at presentation and 2 years following diagnosis were recorded and analysed. In a small number of patients an unacceptable delay in diagnosis was noted. Variation was found in the frequency and type of investigations performed on patients placed on surveillance, types of chemotherapy regimens used and numbers of patients entered into trials. Three per cent of patients had a biopsy of the contralateral testis and 27% of patients defaulted from clinic attendance. Considerable variation in the management of testicular NSGCT in Scotland has been identified. The introduction of management guidelines should result in a more consistent approach to the care of these patients. Support, both financial and psychological, may reduce the unacceptable rate of default.


© 1995 Stockton Press All rights reserved 0007–0920/95 $12.00

### A Scottish national audit of current patterns of management for patients with testicular non-seminomatous germ-cell tumours

GCW Howard[1], K Clarke[2], MH Elia[3], AW Hutcheon[4], SB Kaye[5], PM Windsor[6] and HMA Yosef[7] On behalf of the Scottish Radiological Society and the Scottish Committee of the Royal College of Radiologists

[1]*Department of Clinical Oncology, Western General Hospital, Crewe Road, Edinburgh EH4 2XU;* [2]*Information & Statistics Division, National Health Service in Scotland, Trinity Park House, South Trinity Road, Edinburgh EH5 3SQ;* [3]*Department of Radiotherapy and Oncology, Raigmore Hospital, Inverness IV2 3UJ;* [4]*Department of Medical Oncology, Aberdeen Royal Infirmary, Foresterhill, Aberdeen AB9 2ZB;* [5]*CRC Department of Medical Oncology, Alexander Stone Building, Garscube Estate, Switchback Road, Bearsden, Glasgow G61 1BD;* [6]*Department of Radiotherapy and Oncology, Ninewells Hospital & Medical School, Dundee DD1 9SY;* [7]*Beatson Oncology Centre, Western Infirmary, Glasgow G11 6NT, UK.*

**Summary** A detailed casenote review was performed on all 65 patients registered with testicular non-seminomatous germ cell tumours (NSGCT) during 1989 under the Scottish Cancer Registration Scheme. Details of management at presentation and 2 years following diagnosis were recorded and analysed. In a small number of patients an unacceptable delay in diagnosis was noted. Variation was found in the frequency and type of investigations performed on patients placed on surveillance, types of chemotherapy regimens used and numbers of patients entered into trials. Three per cent of patients had a biopsy of the contralateral testis and 27% of patients defaulted from clinic attendance. Considerable variation in the management of testicular NSGCT in Scotland has been identified. The introduction of management guidelines should result in a more consistent approach to the care of these patients. Support, both financial and psychological, may reduce the unacceptable rate of default.

**Keywords**: audit; non-seminomatous germ cell tumour; management; Scotland; patient compliance

Testicular germ cell tumours are now the commonest cancer in men aged under 40 in Scotland (Sharp *et al.*, 1993*a*). The age-standardised incidence rate of testicular NSGCT in Scotland for 1988–90 was 2.3:100 000, and has risen from 1.8:100 000 for 1975–77 (Sharp *et al.*, 1993*b*). Effective chemotherapy has transformed what was invariably a fatal disease, once it had metastasised, to one which is usually curable (Ellis and Sikora, 1987). Cure rates may be related to the ability to give effective treatment and it has been suggested that results are better when there is a particular expertise in the treatment of the disease (Harding *et al.*, 1993).

Orchidectomy followed by intensive surveillance, which is expensive in clinic and medical time, is now considered the accepted management for most patients with stage I teratoma (Peckham *et al.*, 1982; Horwich, 1993). Patients with poor prognostic histological features, and where the disease has spread beyond the testis, require cytotoxic chemotherapy. Cure rates as high as 90% can now be expected for good prognosis metastatic disease (Horwich, 1989). There are now well-documented prognostic factors that can predict for poorer survival. The role of more intensive chemotherapy regimens for these patients has been the subject of much debate and is currently being assessed by an MRC Trial (Kaye *et al.*, 1989).

The importance of complete surgical excision of residual masses after chemotherapy is now accepted and surgeons experienced in this field may be more likely to perform adequate resections (Ewing *et al.*, 1987; Hendry *et al.*, 1987; Whillis *et al.*, 1991).

Most clinicians continue to follow up intensively patients who have been treated for metastatic NSGCT. The rationale for this is that second-line chemotherapy may be curative if the disease is caught early at the time of relapse. Undoubtedly, some patients may be cured by second-line chemotherapy, but for others salvage therapy should be viewed as palliative (Whillis *et al.*, 1991) and opinions vary as to optimal follow-up for these patients.

Correspondence: GCW Howard
Received 10 January 1995; revised 1 June 1995; accepted 7 June 1995

The aim of this study was to assess variation in investigations at the time of diagnosis and subsequently in the management of patients with both early- and late-stage testicular NSGCT.

### Methods

Details of all testicular NSGCT cases diagnosed between 1 January 1989 and 31 December 1989 were obtained from the Scottish Cancer Registration Scheme. Completeness of registration and validity of diagnosis was checked by cross-referring to oncology centre records and is reported in the accompanying paper (Clarke *et al.*, 1995). A casenote review was performed on all new cancer registrations for testicular NSGCT during 1989. Information was extracted on referral dates, speciality of clinicians involved, staging and pretreatment investigations, histology, prognosis (MRC prognostic group; Mead *et al.*, 1992), treatment and post-treatment follow-up for a 2 year period, patient compliance and any relapse details. Information was recorded onto an agreed proforma and the data anonymised.

Visits and procedures were considered to be part of a formal surveillance policy once an 'active' decision had been documented to pursue such a policy. This was also the definition for post-treatment follow-up, with both being measured for 2 full calendar years from the date of the decision.

### Results

### *Study group*

Sixty-five new cases of testicular NSGCT were registered in Scotland in 1989. Nearly three-quarters of these were aged 34 and under, and only one patient (a paediatric patient treated at a children's hospital who will not be considered further) was not referred to a specialist oncology centre. Two patients (aged 23 and 29) were excluded from the casenote review because of previous primary testicular tumours. Management was therefore not representative of newly diagnosed testicular NSGCT. The numbers of patients registered at each of the five centres A–E respectively were four, four, six, 19 and 29.

In only 39 of 62 cases (62.9%) was the place of initial presentation documented in casenotes. The majority (35 cases, 89.7%) presented to their general practitioner, two to casualty, one to a psychiatric hospital where he had been treated for depression and one to a general surgeon. Date of presentation was not reliably documented so the waiting time for primary referral could not be measured.

Most patients were initially referred to urological (62.9%) or general surgeons (35.5%). Secondary referrals were either to medical oncologists (38.7%), radiation oncologists (48.4%) or urological surgeons (12.9%).

The median wait for a secondary referral was 13 days, range 1–112 days. In four cases the delay was over 8 weeks (71 days, 78 days, 108 days, 112 days). The reasons for these delays were difficult to interpret but in two cases they may have been related to urological waiting lists and the other two patients were misdiagnosed at presentation by general surgeons as having benign disease.

Details of procedures performed at the time of presentation are documented in Table I. All patients had a histological diagnosis of either NSGCT or mixed NSGCT and seminoma.

Staging according to the Marsden system (Peckham *et al.*, 1979) was assigned by the reviewers in all but two cases. Thirty-five (56.5%) were stage I, 11 (17.7%) stage II, one (1.6%) stage III and 13 were stage IV (21.0%). The proportion of patients by stage at presentation varies between centres but this variation is not statistically significant. The statistical test may be unreliable however due to the small numbers involved ($\chi^2 = 19.63$, d.f. = 12, $0.05 < P < 0.10$).

### *Management of stage I disease*

Of 35 patients with stage I disease six (17.1%) received chemotherapy, one (2.9%) received radiotherapy and 28 (80.0%) were placed on surveillance. The six cases receiving chemotherapy were considered to be at high risk of relapse because of known poor risk histological features. Three of these were registered on the MRC high risk stage I trial. The single case that received radiotherapy was initially diagnosed as a seminoma.

A surveillance policy was pursued in 28 patients. One died in a road traffic accident during the first year and seven cases relapsed (25.0%). Seven of the remainder (35.0%) had defaults documented in their notes. A patient was considered to have defaulted if he had missed at least one appointment.

Patients on surveillance excluding defaulters were seen between eight and 15 times (median 12) during the first year and three and seven times (median six) during the second year. At each visit tumour markers alphafetoprotein (AFP) and human chorionic gonadotrophin (HCG) were performed, plus a chest radiograph if a computerised tomography (CT) scan had not been booked around the time of the visit. CT scans of the thorax and abdomen were performed on zero to six occasions (median four) in the first year, and zero to three times (median two) in the second year (Table II). Ultrasound was not a procedure recorded but some reviewers noted it had been included in the surveillance programme in a small number of cases.

Seven (25%) stage I cases relapsed on surveillance, all within 6 months of diagnosis (median 17 weeks, range 7–22 weeks).

### *Treatment of patients with metastatic disease*

Thirty-four patients were treated for metastatic disease, of which seven were relapsed stage I cases. All cases received chemotherapy. Four chemotherapy regimens were used for patients with good prognosis disease, with 10 of 20 such patients treated within a clinical trial setting. Six regimens were used for patients with poor prognostic disease with seven of the 12 treated within a trial setting. A single regimen was used for the two patients with an unknown prognosis.

During primary therapy only one patient received radiotherapy. This patient received ten courses of combination chemotherapy followed by whole brain radiotherapy (4048 cGy in 22 daily fractions over 33 days).

At the end of primary therapy 18 cases (52.9%) had a complete regression (CR), eleven (32.4%) had partial regression (PR), one had progressive disease, in three cases the status was inevaluable (markers normal, no or equivocal CT scans) and in a further case there was no information.

Sixteen patients did not achieve complete remission at the end of therapy. Three patients had no further treatment, the rest having residual masses excised with or without further chemotherapy. Eleven of the 12 patients who had surgery achieved a CR.

Defaults from follow-up were recorded in 35% of patients on active surveillance and 21% of patients with post-treatment follow-up (27% for all clinic attendances). The commonest reasons recorded for defaulting were anxiety, transport difficulties and lack of finance.

There was a marked difference in the number of patients being treated in a trial setting at a time when almost all patients would have been eligible for inclusion within MRC studies. Only two of the five centres entered patients with 69% and 53% of all patients being entered by centres E and D respectively.

**Table I** Procedures performed at staging and pretreatment

| *Procedure* | *Performed (as documented in notes)* *Yes* | *No* | *Not known* | *Percentage performed* |
|---|---|---|---|---|
| Pre-operative AFP | 51 | 6 | 5 | 82 |
| Pre-operative HCG | 51 | 6 | 5 | 82 |
| Pre-operative LDH | 10 | 21 | 31 | 16 |
| Post-operative AFP | 60 | 0 | 2 | 97 |
| Post-operative HCG | 60 | 0 | 2 | 97 |
| Post-operative LDH | 18 | 14 | 30 | 29 |
| Ultrasound testis | 25 | 29 | 8 | 40 |
| Ultrasound abdomen | 14 | 35 | 13 | 23 |
| CT thorax | 58 | 1 | 3 | 94 |
| CT abdomen | 61 | 0 | 1 | 98 |
| CT brain | 1 | 56 | 5 | 2 |
| Chest radiograph | 52 | 4 | 6 | 84 |
| MRI | 3 | 56 | 3 | 5 |
| Bipedal lymphography | 6 | 52 | 4 | 10 |
| CSF markers | 0 | 57 | 5 | 0 |
| Biopsy contralateral testis | 2 | 58 | 2 | 3 |
| Pathology review | 30 | 27 | 5 | 48 |
| Sperm count and or storage | 14 | 36 | 12 | 23 |
| Other | 6 | 54 | 2 | 10 |

**Table II** Frequency of attendance and procedures performed on stage I NSGCT patients on surveillance (excluding defaulters)

| | *Number of attendances or procedures* | | | | | | | | | | | | | | | | | |
|---|---|---|---|---|---|---|---|---|---|---|---|---|---|---|---|---|---|---|
| | *0* | *1* | *2* | *3* | *4* | *5* | *6* | *7* | *8* | *9* | *10* | *11* | *12* | *13* | *14* | *15* | *NK* | *Total* |
| *Year 1* | | | | | | | | | | | | | | | | | | |
| Visits | 0 | 0 | 0 | 0 | 0 | 0 | 0 | 0 | 1 | 0 | 3 | 0 | 5 | 1 | 2 | 1 | 0 | 13 |
| AFP | 0 | 0 | 0 | 0 | 0 | 0 | 0 | 0 | 1 | 1 | 1 | 1 | 5 | 1 | 3 | 0 | 0 | 13 |
| HCG | 0 | 0 | 0 | 0 | 0 | 0 | 0 | 0 | 1 | 1 | 1 | 1 | 5 | 1 | 3 | 0 | 0 | 13 |
| LDH | 5 | 0 | 0 | 0 | 0 | 0 | 0 | 0 | 0 | 1 | 1 | 0 | 0 | 1 | 0 | 0 | 5 | 13 |
| Chest radiograph | 0 | 0 | 0 | 0 | 1 | 0 | 2 | 3 | 1 | 2 | 3 | 0 | 1 | 0 | 0 | 0 | 0 | 13 |
| CT thorax | 3 | 0 | 0 | 2 | 3 | 2 | 3 | 0 | 0 | 0 | 0 | 0 | 0 | 0 | 0 | 0 | 0 | 13 |
| CT abdomen | 1 | 0 | 2 | 2 | 2 | 2 | 4 | 0 | 0 | 0 | 0 | 0 | 0 | 0 | 0 | 0 | 0 | 13 |
| *Year 2* | | | | | | | | | | | | | | | | | | |
| Visits | 0 | 0 | 0 | 1 | 2 | 3 | 6 | 1 | 0 | 0 | 0 | 0 | 0 | 0 | 0 | 0 | 0 | 13 |
| AFP | 0 | 0 | 0 | 1 | 2 | 3 | 6 | 1 | 0 | 0 | 0 | 0 | 0 | 0 | 0 | 0 | 0 | 13 |
| HCG | 0 | 0 | 0 | 1 | 2 | 3 | 6 | 1 | 0 | 0 | 0 | 0 | 0 | 0 | 0 | 0 | 0 | 13 |
| LDH | 5 | 0 | 0 | 1 | 1 | 0 | 1 | 0 | 0 | 0 | 0 | 0 | 0 | 0 | 0 | 0 | 5 | 13 |
| Chest radiograph | 0 | 1 | 0 | 2 | 6 | 2 | 2 | 0 | 0 | 0 | 0 | 0 | 0 | 0 | 0 | 0 | 0 | 13 |
| CT thorax | 3 | 2 | 7 | 1 | 0 | 0 | 0 | 0 | 0 | 0 | 0 | 0 | 0 | 0 | 0 | 0 | 0 | 13 |
| CT abdomen | 3 | 2 | 7 | 1 | 0 | 0 | 0 | 0 | 0 | 0 | 0 | 0 | 0 | 0 | 0 | 0 | 0 | 13 |

NK, not known.

### Discussion

Documentation regarding the patient's first presentation was poor (only recorded for 63% of cases), but most presented to their general practitioner and were referred to either a general or urological surgeon. Secondary referral to oncologists or urological surgeons involved a median wait of 13 days (range 1–112 days) with four cases having delays of over 8 weeks. Although the reasons for such delays are not easy to interpret it would appear that in two cases this was related to urology waiting times. In the other two cases this was related to an initial diagnosis of benign disease.

The documentation of staging and pretreatment investigations varied considerably between cases with the frequency of procedures probably being underrecorded.

Although many clinicians advocate biopsy of the contralateral testis in all patients to exclude carcinoma *in situ* (Hargreaves, 1986), this was only performed in 3% of cases. This highlights the reluctance of surgeons to inflict another invasive procedure on their patients and the controversy surrounding the necessity for biopsy. Investigations performed at presentation for diagnosis and staging are predictable. Lactate dehydrogenase is rarely measured, which illustrates the controversy as to how valuable this is. Although a non-specific marker, high levels are indicative of a poor prognosis and it may be the only marker in seminoma. As a general rule there is no role for abdominal ultrasound now that CT scanning is widely available. MRI has only recently been introduced and it is not clear as to whether it has any advantage over CT in the management of the disease.

Facilities for sperm storage are available in four of the five centres. Centre A is the exceptions sending sample, to centre B for storage. This was not performed in all patients receiving chemotherapy. There may be good but undocumented reasons for this (e.g. previous vasectomy or clinical urgency to commence treatment). Although the majority of patients with normal sperm counts before therapy will regain fertility after chemotherapy, facilities for storage of sperm should be readily accessible for all patients.

There is considerable variation in the frequency of follow-up and investigations performed when patients are managed on an active surveillance policy. Although there will be many reasons for increasing the frequency of investigations in specific patients there should be a minimum number of attendances and investigations which is considered to be good practice. The variation in CT scanning of the abdomen between zero and six times and zero and three times in the first and second year respectively is of concern.

The incidence for defaulting from surveillance and post-treatment follow-up is worryingly high with 35.0% and 21.4% of cases defaulting respectively. Patients on surveillance in another study have been found to be less compliant compared with those having received chemotherapy as they underestimated the need for follow-up (Young *et al*., 1991). It is therefore important that only those who are reliable are selected for active surveillance. Some assistance in the form of transport or counselling regarding the importance of follow-up may be necessary.

Chemotherapy regimens for both poor and good risk patients with metastatic disease were largely predictable. Four different regimens for good prognosis disease were used, all are well recognised but vary considerably in their toxicity. Such variation should not exist outside clinical trials and it is noteworthy that only 10 of 20 patients were treated in a trial setting. There should be a consensus as to the most appropriate first-line therapy unless patients are entered into a trial. Six regimens were used for patients with poor prognostic disease with seven out of 12 in trials. Only two of the five centres put patients into clinical trials. The three centres which did not participate stated that this was due to lack of clerical support in 1989, but with the recent introduction of the Scottish Cancer Therapy Network (SCTN) this issue has already been addressed.

The importance of complete resection of residual masses after chemotherapy is well documented. In this study a total of 12 operations were undertaken in a single year by three urological surgeons, one cardiothoracic and two general surgeons in three centres. It may be that with this level of workload such surgery should be performed by fewer surgeons.

Referral patterns in general were predictable but unacceptable delays occurred in a small number of cases awaiting surgery. In at least two cases this was due to the patients being diagnosed as having benign disease and to avoid such delays there should be a high index of suspicion that patients with testicular symptoms might have cancer and are treated urgently.

This study has highlighted considerable variation in several different aspects of the management of NSGCT in Scotland. Follow-up policies for patients on surveillance as well as those after chemotherapy varied considerably and guidelines should be laid down as to what is the minimum advisable with regard to clinic appointments and investigations. Defaults from surveillance and follow-up occurred too often and arrangements for transport or other financial support and psychological support should be made available.

Multiple chemotherapy regimens were used for patients with both good and poor prognostic disease. Although there will always be reasons for tailoring chemotherapy to individual patients national guidelines for first- and second-line chemotherapy for patients not entered into a trial protocol might result in a more consistent approach to the management of this disease.

The trial entry is poor for a rare tumour at a time when almost all patients would have been eligible for one trial or another. There is a particularly noticeable variation in trial entry between centres. The reasons for this relate to lack of clerical and other support and this should improve following the setting up of the Scottish Cancer Therapy Network.

**Acknowledgements**

We wish to thank all consultants who assisted this project by allowing access to their patients, casenotes and/or radiology and to associated medical records officers and radiology departments for making them available; Mrs Mary Jack, Miss Gill Kerr, Mrs Myrtle Adams, Mrs Liz Smart and Ms Karen McGregor for their assistance with records at oncology centres; Dr Calum Muir, Ms Linda Sharp, Ms Jan Warner, Dr John Clarke and Mr Steve Kendrick of Information and Statistics Division for providing data; and Ms Linda Sharp for statistical assistance. This work was funded by the Clinical and Resource and Audit Group of the Scottish Office.

**References**

CLARKE K, HOWARD GCW, ELIA MH, HUTCHEON AW, KAYE SB, WINDSOR PM AND YOSEF HMA. (1995). A Scottish national audit of referral patterns to specialist oncology centres for patients with testicular germ cell tumours. *Br. J. Cancer*, **72**, 1300-1302.

ELLIS M AND SIKORA K. (1987). The current management of testicular cancer. *Br. J. Urol.*, **59**, 2-9.

EWING R, HETHERINGTON JW, JONES WG AND WILLIAMS RE. (1987). Surgical salvage of advanced testicular tumours. *Br. J. Urol.*, **59**, 76-80.

HARDING MJ, PAUL J, GILLIS CR AND KAYE SB. (1993). Management of malignant teratoma: does referral to a specialist unit matter? *Lancet*, **1**, 999-1002.

HARGREAVES TB. (1986). Carcinoma in situ of the testis. *Br. Med. J.*, **293**, 1389-1390.

HENDRY WF, GOLDSTRAW P AND PECKHAM MJ. (1987). The role of surgery in the combined management of metastases from malignant teratoma of the testes. *Br. J. Urol.*, **59**, 358.

HORWICH A. (1989). Germ cell tumour chemotherapy. *Br. J. Cancer*, **59**, 156-159.

HORWICH A. (1993). Current issues in the management of clinical stage I testicular teratoma. *Eur. J. Cancer*, **29A**, 933-934.

KAYE SB, HARDING M, STOKER G, FOSSA S. *et al.* (1989). 'BOP-VIP' – a new intensive regime for poor prognosis germ cell tumours. *Proc. Am. Soc. Clin. Oncol.*, **8**, 136.

MEAD GM, STENNING SP, PARKINSON MC, HORWICH A, FOSSA SD, WILKINSON PM, KAYE SB, NEWLANDS ES AND COOK PA. (1992). The second Medical Research Council Study of prognostic factors in non-seminomatous germ cell tumours. *J. Clin. Oncol.*, **10**, 85-94.

PECKHAM MJ, McELWAIN TJ, BARRETT A AND HENDRY WF. (1979). Combined management of teratoma of the testis. *Lancet*, **2**, 267-270.

PECKHAM MJ, BARRETT A, HUSBAND JE AND HENDRY WF. (1982). Orchidectomy alone in testicular stage I non-seminomatous germ-cell tumours. *Lancet*, **2**, 678-680.

SHARP L, BLACK RJ, HARKNESS EF, FINLAYSON AR AND MUIR CS. (1993*a*). *Cancer Registration Statistics Scotland 1981-1990.* Information and Statistics Division: Edinburgh.

SHARP L, BLACK RJ, MUIR CS, WARNER J AND CLARKE JA. (1993*b*). Trends in cancer of the testis in Scotland, 1961-90. *Health Bull.*, **51**, 255-267.

WHILLIS D, COLEMAN RE, LESSELLS AM, HARGREAVE TB, CORNBLEET MA AND HOWARD GCW. (1991). Surgery following chemotherapy for metastatic testicular teratoma. *Br. J. Urol.*, **68**, 292-295.

WHILLIS D, COLEMAN RE, CORNBLEET MA AND HOWARD GCW. (1991). Failure of salvage treatment in metastatic testicular teratoma. *J. Clin. Oncol.*, **3**, 141-146.

YOUNG BJ, BULLTZ JA AND TREW MS. (1991). Compliance with follow-up of patients treated for non-seminomatous testicular cancer. *Br. J. Cancer*, **64**, 606-608.